# Pulmonary NUT carcinoma, an elusive and refractory entity, shows transient response to chemotherapeutics and PD-1 inhibitor: a case report and literature review

**DOI:** 10.3389/fimmu.2025.1497124

**Published:** 2025-03-11

**Authors:** Guangjian Yang, Runze Liu, Linke Yang, Xue Yang, Xiaoyong Tang, Huiqing Mao

**Affiliations:** ^1^ Department of Respiratory Medical Oncology, Shandong Cancer Hospital and Institute, Shandong First Medical University and Shandong Academy of Medical Sciences, Jinan, Shandong, China; ^2^ Department of Radiation Oncology, Shandong Cancer Hospital and Institute, Shandong First Medical University and Shandong Academy of Medical Sciences, Jinan, Shandong, China; ^3^ Department of Pathology, Shandong Cancer Hospital and Institute, Shandong First Medical University and Shandong Academy of Medical Sciences, Jinan, Shandong, China

**Keywords:** NUT carcinoma, pulmonary, chemotherapy, PD-1 inhibitor, case report

## Abstract

Nuclear protein of the testis (NUT) carcinoma (NC) is a rare but highly aggressive disease, characterized by drug resistance and poor prognosis. This report describes the case of a 32-year-old male patient diagnosed to have pulmonary NC; the tumor exhibited positive immunohistochemical staining of NUT and showed rearrangement of BRD4::NUT midline carcinoma family member 1 (NUTM1). After two treatment cycles of chemotherapy (etoposide plus carboplatin) combined with the PD-1 inhibitor sintilimab, the thoracic lesion of the patient disappeared, resulting in a partial response. When the patient’s disease progressed even after the targeted therapy with a bromodomain and extra-terminal motif (BET) inhibitor, sintilimab was readministered in combination with platinum-based chemotherapy. However, the disease rapidly progressed after only one treatment cycle. Notably, the disease showed *de novo* drug resistance to the combination of chemotherapy with the histone deacetylase inhibitor. Although the patient’s NC initially responded well to the combination of the PD-1 inhibitor and chemotherapy, the response was transient. These findings suggest that pulmonary NC is a highly malignant thoracic carcinoma, with no durable response and survival benefits from treatment with chemotherapeutics or immune checkpoint inhibitors.

## Introduction

Nuclear protein of the testis (NUT) carcinoma (NC) is a rare and undifferentiated malignancy primarily observed in young adults and children, and it is characterized by highly aggressive behavior and poor prognosis (1–3). The most common location of primary NC is the thorax (50%), followed by the head and neck region (40%) (4). NC is genetically defined by chromosomal translocation t (15;19) between the NUT midline carcinoma family member 1 (*NUTM1*) gene and the bromodomain (*BRD*) family members *BRD3* or *BRD4* gene, which results in the BRD3::NUTM1 or BRD4::NUTM1 fusion oncogene (5). Most patients with NC typically develop extensive disease at the initial diagnosis stage and progress rapidly to death, with a median overall survival (OS) of 6.7 months (1). The 2015 World Health Organization (WHO) classification initially added NC to a category of other and unclassified lung tumors, and defined it to be associated with the presence of *NUTM1* gene rearrangement (6). Additionally, nonthoracic NC with non-BRD4::NUTM1 fusion shows the best prognosis, followed by nonthoracic NC cases with BRD4::NUTM1. Thoracic NC patients have the worst survival rate, with a median OS of only 4.4 months and a 2-year OS of 5%, regardless of the *NUT* fusion variants (4). Here, we present the case of a patient with pulmonary NC with BRD4::NUTM1 fusion, who underwent multiple therapeutic interventions and achieved an OS of 15 months.

## Case presentation

**Figure 1:**
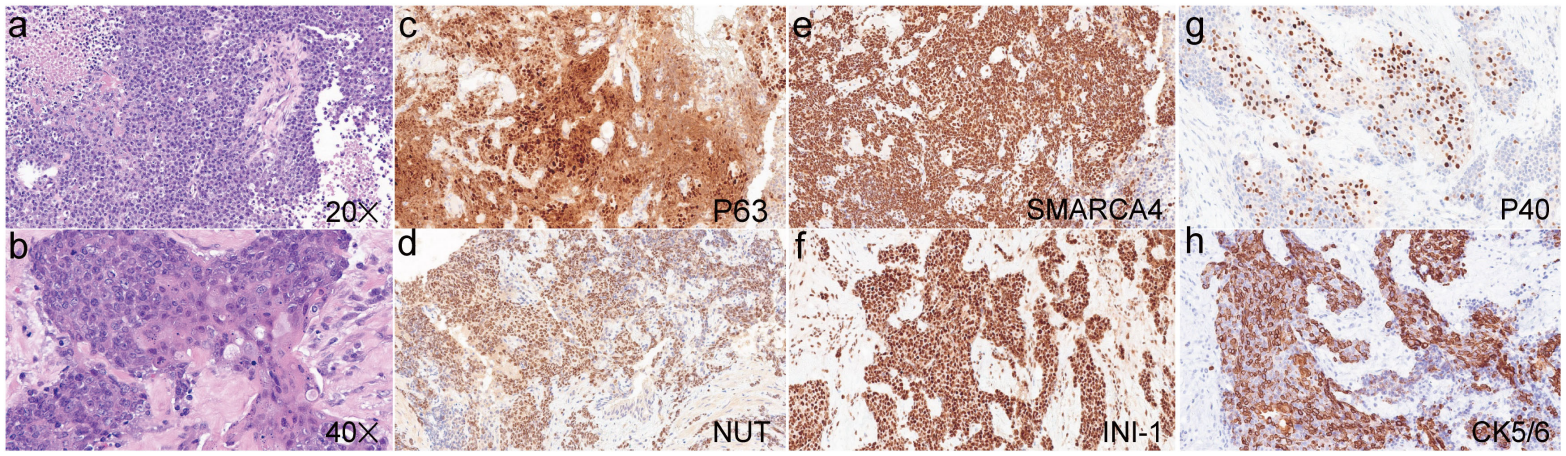
Hematoxylin and eosin staining of the pulmonary NUT carcinoma (**a**, ×20; **b**, ×40). Positive immunohistochemical staining was observed for P63 **(c)**, NUT **(d)**, SMARCA4 **(e)**, INI-1 **(f)**, P40 **(g)** and CK5/6 **(h)**.

**Figure 2:**
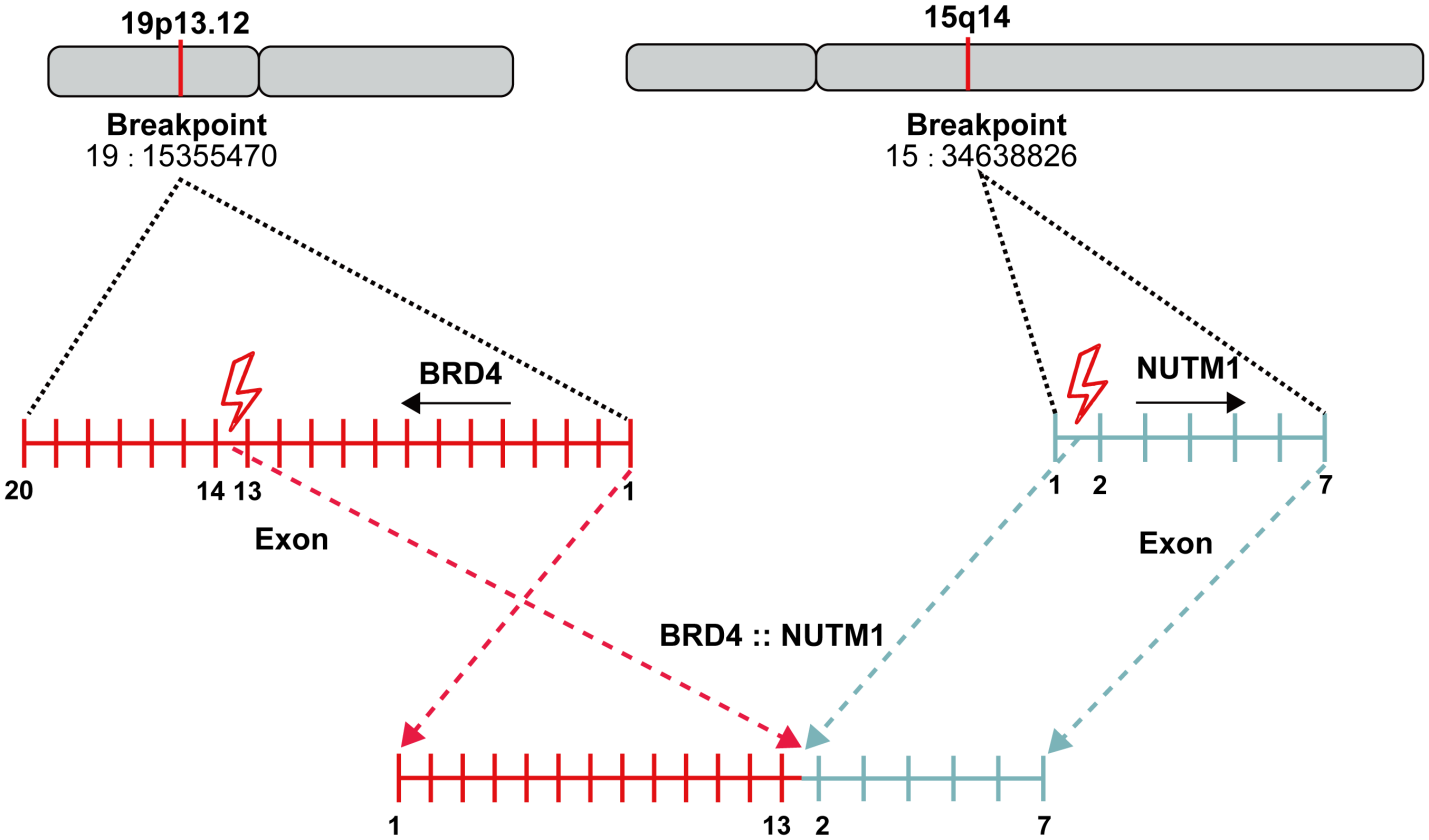
Genomic structure of BRD4 (exon 13)::NUTM1 (exon 2) translocation in this case.

A 32-year-old male with no smoking history was diagnosed to have lung cancer in October 2022. Positron emission tomography-computed tomography (PET-CT) examination revealed a lesion (2.9×1.8cm) in the lower lobe of the left lung, with multiple mediastinal lymph node and bone metastases in the pubis, scapula, ilium, ribs and lumbar vertebra. Subsequent transbronchial needle aspiration revealed nests of small-sized to intermediate-sized poorly differentiated tumor cells in microscopy examination. The nuclei exhibited atypical features, including irregular contours and granular to coarse chromatin (**Figures 1a, b**). Immunohistochemical (IHC) staining was performed for a comprehensive panel of biomarkers. Positive staining was observed for P63 (**Figure 1c**), NUT (**Figure 1d**), SMARCA4 (**Figure 1e**), INI-1 (**Figure 1f**), P40 (**Figure 1g**) and CK5/6 (**Figure 1h**), and negative staining was noted for programmed cell death-ligand 1 (PD-L1). Subsequent next-generation sequencing (NGS) using the GeneseeqPrime™ kit containing a target panel of 437 genes confirmed the presence of BRD4 (exon 13)::NUTM1 (exon 2) fusion (**Figure 2**), along with other missence alterations of uncertain significance in the genes *EPHA3*, *LRP1B*, *MECOM*, and *PKHD1*.

**Figure 3:**
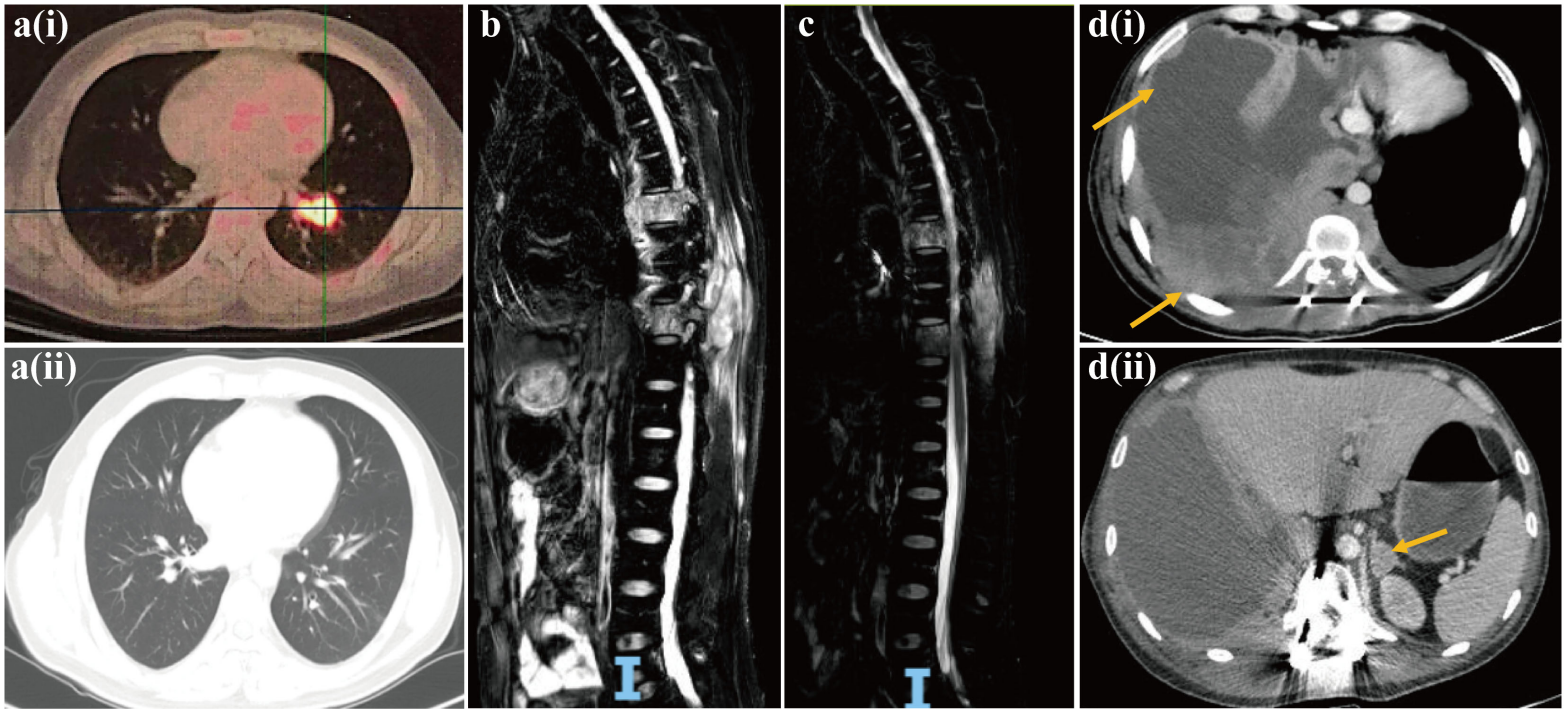
Computed tomography images of pulmonary NUT carcinoma pre-[**a(i)**] and post [**a(ii)**] two treatment cycles of chemotherapy (etoposide plus carboplatin) in combination of PD-1 inhibitor sintilimab. Magnetic resonance image of progressive disease with multiple bone and soft tissue metastatic lesions, and worsening of bone destruction after 4 months of treatment with BET inhibitor **(b)**. Magnetic resonance image of reduction in multiple soft tissue lesions, and the improvement of spinal stenosis at the seventh and tenth thoracic vertebrae following chemotherapy in combination of anti-angiogenesis agent bevacizumab and radiotherapy **(c)**. Computed tomography images of multiple lesions developing in the pleura, chest wall [**d(i)**] and adrenal gland [**d(ii)**].

The patient exhibited a favorable functional status (score of 0) according to the Eastern Cooperative Oncology Group Performance Status (ECOG PS) scale, and started receiving chemotherapy (etoposide plus carboplatin) combined with the anti-PD-1 inhibitor sintilimab from December 2022. After two treatment cycles, a chest and abdominal CT scan on February 14, 2023, revealed reduced mediastinal lymph nodes and stable status of bone metastases in the pubis, scapula, ilium, ribs and lumbar vertebra. The lesion diagnosed in the lower lobe of the left lung at baseline diagnosis had resolved after two treatment cycles, and the efficacy was evaluated as a partial response (**Figure 3a**). Despite stable bone metastases, the patient experienced gradual aggravation of bone pain and discontinued his chemotherapy. In February 2023, the patient’s ECOG PS was still 0, and he was enrolled in a phase I clinical study for patients with advanced solid tumors or lymphoma (ChiCTR2000040198). Subsequently, he received the bromodomain and extra-terminal motif inhibitor (BET-i) “NHWD-870 HCl” for targeted therapy. This treatment significantly alleviated his severe bone pain, and a CT scan in April 2023 revealed stable disease in both thoracic and bone metastases. Four months later, examination by magnetic resonance imaging (MRI) showed multiple bone and soft tissue metastatic lesions, with worsening of bone destruction and increased bone pain. The disease progressed in June 2023 after 4 months of treatment with BET-i (**Figure 3b**).

In July 2023, the patient’s ECOG PS remained at 0, and he underwent another round of chemotherapy (albumin-bound paclitaxel plus carboplatin) in combination with the anti-angiogenesis agent bevacizumab. Concurrently, he received radiotherapy for vertebral tumors at the dose of 40Gy/4Gy/10f. An MRI examination in August 2023 revealed a reduction in multiple soft tissue lesions, and the spinal stenosis at the seventh and tenth thoracic vertebrae showed improvement (**Figure 3c**). However, in September 2023, the bone and soft tissue metastatic lesions progressed again, along with worsening of spinal stenosis. Given the partial response after only two cycles of first-line chemotherapy with sintilimab, and based on his favorable ECOG PS score of 0 and discontinuation of the regimen due to the gradual aggravation of bone pain rather than due to disease progression, the medical team recommended rechallenge with first-line chemotherapy (etoposide plus carboplatin) combined with sintilimab. Unfortunately, after only one treatment cycle, the patient developed neurothlipsis, leading to paraplegia due to the refractory spinal metastasis. Subsequently, in November 2023, decompression and excision of spinal canal lesions were performed. The postoperative pathology confirmed metastatic NC, with positively stained NUT in IHC analysis.

**Figure 4:**
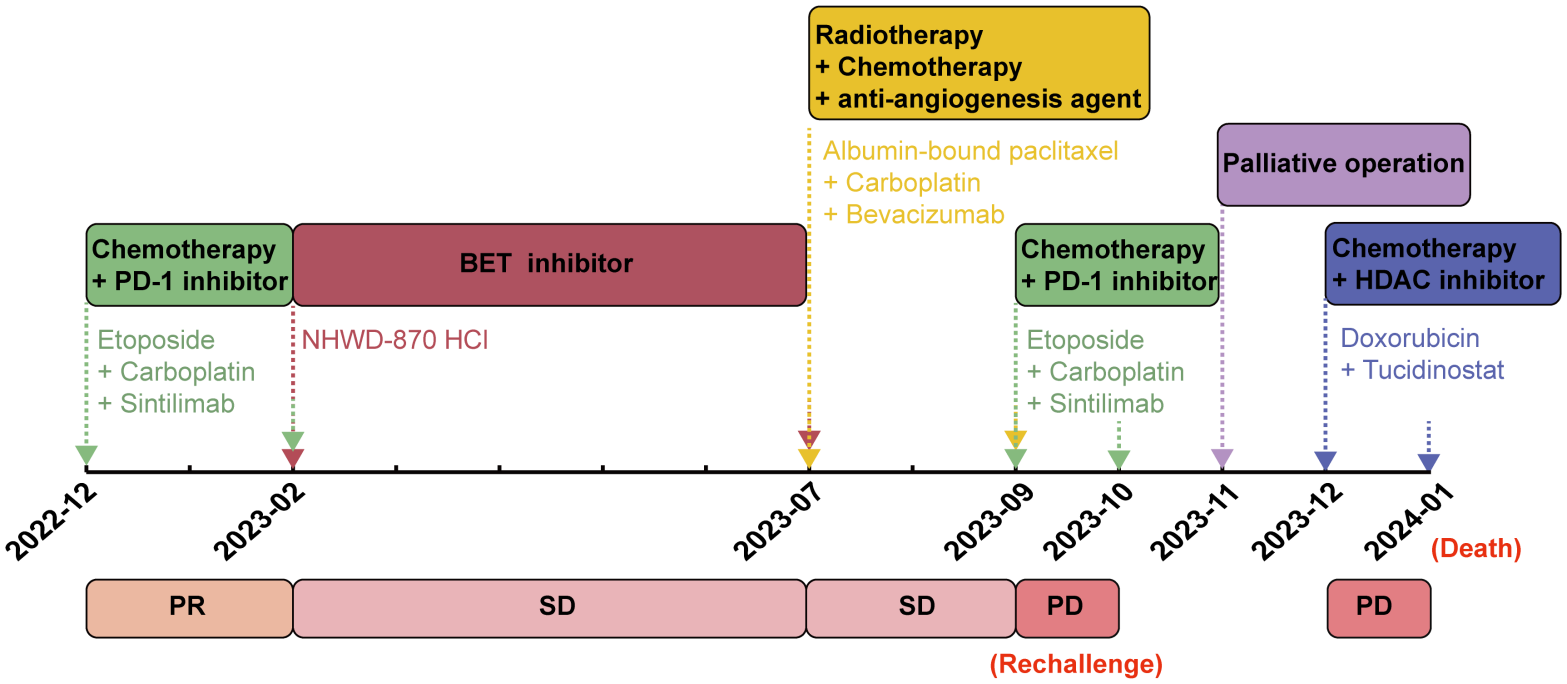
The timeline of treatment in this present case.

In December 2023, the patient’s functional status deteriorated (ECOG PS score: 2). He received single-agent chemotherapy with doxorubicin, combined with the histone deacetylase inhibitor (HDAC-i) tucidinostat for epigenetic therapy. Unfortunately, his health condition rapidly worsened, with multiple lesions developing in the pleura, chest wall and adrenal gland (**Figure 3d**). Finally, in January 2024, the patient succumbed to acute respiratory failure and dyscrasia, with an OS period of 15 months. The timeline of this patient’s treatment was depicted in **Figure 4**.

## Discussion

Currently, more than 100 cases of NC have been reported worldwide. The NC registry, which is the largest sample-sized study on NC to date, reported 141 patients from 17 countries between 1993 and 2017 (4). This comprehensive analysis investigated the clinical and molecular characteristics of NC, including the median age at diagnosis (23.6 years, range: 18 days to 80 years), gender distribution (52% female), tumor locations (thorax: 51%, head and neck: 41%, bone and soft tissue: 6%, and kidney: 1%), *NUTM1* fusion partners (78% BRD4::NUTM1, 15% BRD3::NUTM1, and 6% NSD3::NUTM1), and histological types (54% carcinoma without squamous differentiation and 33% carcinoma with squamous differentiation) (4). Although NC was originally thought to be a disease primarily affecting children and young adults with a median age of 24 years, NC can occur in individuals of any age, and equally affects males and females (1, 4). Regarding molecular characterization, approximately three-fourth of NC cases exhibit BRD4::NUTM1 fusion, and the remaining NC cases show less common *NUTM1* fusion partners, including *BRD3* and *NSD3* (7). NC typically presents as nests of monomorphic small-sized to medium-sized undifferentiated round cells, with abundant necrosis, numerous neutrophils in the background, and high mitotic rates. These features can lead to misdiagnosis as small cell lung cancer, squamous cell carcinoma, thymic carcinoma, or Ewing’s sarcoma (8, 9). Focal squamous differentiation with abrupt keratinization is considered a hallmark of NC, although nearly 54% of NC cases lack squamous cell differentiation (4, 6). Diagnosis of NC can be achieved through IHC analysis using a monoclonal antibodies against NUT, or confirmed directly by the presence of *NUTM1* fusion through FISH, and DNA or RNA sequencing (10–12).

NC typically exhibits nuclear IHC markers of squamous differentiation, such as P63 and P40, along with keratins. However, this squamous differentiation often complicates the accurate diagnosis of NC, leading to frequent misdiagnosis as squamous cell carcinoma or other tumor types. NUT-specific monoclonal antibodies demonstrated 100% specificity and 87% sensitivity for NC diagnosis, thereby making positive NUT IHC staining a direct and reliable indicator of NC presence (10). In the present case, the poorly differentiated NC positively expressed keratin, P63, P40, and NUT. Additionally, BRD4::NUTM1 fusion was identified through NGS, which further corroborates the pathological characteristics of NC discussed above.

The largest sample-sized study to date revealed a median OS of 6.5 months for NC, confirming its poor prognosis consistent with previous findings (1, 4). Currently, no standard management exists for pulmonary NC. While radical resection or chemoradiotherapy can temporarily control the disease in some NC patients, their average OS is still very short, with no chemotherapeutic agents demonstrating survival benefits (1, 13–16). Long-term survival of NC patients has been attributed to the early complete surgical resection or initial radiotherapy in some cases (1, 13). Previous studies have shown that radiotherapy is effective for NC originating in the head, neck and lungs, but not for NC with mediastinal primary origin (15). In the absence of a standard chemotherapy regimen for NC, platinum-based chemotherapy with paclitaxel or gemcitabine is commonly used clinically for its broad spectrum antitumor activity. Additional chemotherapeutic options include doxorubicin, actinomycin D, anthracycline, docetaxel, etoposide, vincristine, vinorelbine, cyclophosphamide, and ifosfamide (15). Beesley et al. observed that vincristine significantly reduced the tumor burden in NC xenografts, but failed to prevent tumor recurrence (17). In the present case, despite the initial favorable response of the patient to platinum-based chemotherapy with etoposide or albumin-bound paclitaxel, he rapidly developed progressive disease after several treatment cycles. Furthermore, although the patient initially showed a transient response to the PD-1 inhibitor, the disease quickly progressed after only one treatment cycle when immunotherapy was reintroduced in the later-line setting.

The BRD4::NUTM1 fusion can activate *EP300*, contributing to an aggressive phenotype by promoting cell growth and inhibiting differentiation through aberrant histone acetylation (8, 18, 19). As a key inhibitor of tumor cell differentiation and growth HDAC-i restores chromatin acetylation and induces differentiation of NC cells, thus showing potential benefits in treating NC (9). BET-i, acting as acetyl histone mimetics, specifically binds to the BET bromodomain and competitively inhibits its binding to the chromatin (20). The antitumor effect of BET-i has also been confirmed in NC (21, 22). Several BET-i, including OTX105/MK-8628, GSK525762, BAY1238097, GSK2820151, and TEN-010, are currently in clinical trials for NC treatment (20, 21, 23). Notably, BET-i treatment has shown promising results, demonstrating decreased *NUT* expression in NC (24). JQ1 and OTX105/MK-8628 (birabresib), two representative BET-i, have exhibited antitumor effects in both patients and xenograft models of NC (23, 25, 26). Additionally, the cyclin-dependent kinase 4/6 (CDK4/6) inhibitors have shown synergistic effects with BET-i in NC, thus presenting a potential targeted option (27). In the present case, the patient experienced rapid disease progression after 4-month treatment of BET-i, and demonstrated *de novo* resistance to doxorubicin combined with HDAC-i tucidinostat. Although HDAC-i and BET-i have emerged as two promising targeted agents for NC, their accessibility, efficacy, toxicity and survival benefits in these patients remain significant challenges for future research.

## Conclusions

In conclusion, pulmonary NC represents a rare yet highly aggressive thoracic malignancy, characterized by transient response to the combination of chemotherapy and PD-1 inhibitor, and a poor overall prognosis. Epigenetic agents, including HDAC and BET-i, offer promising avenues for targeted therapy in NC management.

## Data Availability

Data presented in this case is included in article, and will be made available from the corresponding author upon reasonable request.
